# German and English Literature

**Published:** 1892-06

**Authors:** 


### GERMAN AND ENGLISH LITERATURE.

As an indication of Shakespeare's popularity in Germany it is said that 17,000 copies of a cheap edition of his works were sold in the Fatherland last year. It would be curious to know how many copies of Goethe are bought by Americans annually. The Germans have always been more deeply interested in our great writers than we have been in theirs, and Macaulay said that our great-grandfathers did not even know of the existence of German literature. The most extensive English grammar, Maetzner's is the work of a German, and the first great authorities in Anglo-Saxon and Early English were the German scholars Maetzner and Grein, now dead. The greatest authority in English phonology, not excepting Ellis and Sweet, is Edouard Sievers, a young Jena professor whom Harvard once tried to get. Of very recent years there has been much creditable work in Anglo-Saxon by American professors. Within the last five years there have been no fewer than four editions of the Anglo-Saxon poem "Beowulf" in America and two in England, yet as late as ten years ago the only respectable edition of that poem in existence was by a German.

